# ARTEM: a method for RNA and DNA tertiary motif identification with backbone permutations

**DOI:** 10.1186/s13059-025-03696-2

**Published:** 2025-07-28

**Authors:** Eugene F. Baulin, Davyd R. Bohdan, Dawid Kowalski, Milena Serwatka, Julia Świerczyńska, Zuzanna Żyra, Janusz M. Bujnicki

**Affiliations:** 1 International Institute of Molecular and Cell Biology in Warsaw, Ul. Ks. Trojdena 4, 02-109 Warsaw, Poland; 2 IMol Polish Academy of Sciences, Ul. M. Flisa 6, 02-247 Warsaw, Poland; 3 https://x.com/febos41; 4 University of Warsaw, Faculty of Medicine, Ul. Żwirki I Wigury 101, 02-089 Warsaw, Poland; 5 Military Institute of Medicine - National Research Institute, Szaserow 128, 04-141 Warsaw, Poland; 6 Medical University of Gdańsk, Ul. Marii Skłodowskiej-Curie 3a, 80-210 Gdańsk, Poland

**Keywords:** Kink-turn, K-junction, A-minor junction, Tertiary motif, Nucleic acid structure, Motif search, Backbone topology, Isostericity

## Abstract

Non-coding RNA functions are largely defined by their 3D structures, which consist of recurrent building blocks, tertiary motifs. The computational motif search problem remains largely unsolved, as standard approaches are restrained by sequence, interactions, or backbone topology. We present ARTEM 2.0, which enables automated, unrestrained searches of RNA and DNA structure databases to identify 3D motifs. We apply ARTEM for searching kink-turns, G-quadruplexes, GNRA tetraloops, and i-motifs. ARTEM outperforms existing methods and enables the discovery of novel motif variants. ARTEM opens a fundamentally new way of studying nucleic acid 3D folds and motifs and analyzing their correlations and variations.

**Supplementary Information:**

The online version contains supplementary material available at 10.1186/s13059-025-03696-2.

## Background

The functions of non-coding RNAs are largely defined by their three-dimensional structures [1]. RNA 3D structure is organized hierarchically and can be seen as a set of building blocks, RNA 3D modules [2]. The recurrent modules that preserve their features in various RNAs across different structural contexts are called RNA tertiary motifs [3]. Commonly, RNA tertiary motifs are classified into two major classes: local loop motifs, nested within elements of RNA secondary structure [4], such as kink-turns [5], and long-range motifs formed between distant structural segments, such as D-loop/T-loop interaction motifs [6]. Long-range motifs remain much less explored [7, 8] compared to loop motifs [4].

Among the main reasons for the lag in long-range motif exploration are the limitations of existing computational methods designed for the motif search problem, which is formulated as the task of identifying instances of a given 3D motif within a given 3D structure or a set of structures [9–14]. The commonly used methods are limited by at least one of three major restraints: sequence (types of bases) [10, 11], annotations of interactions (e.g., types of base pairs [15]) [9, 11, 12, 14], and backbone topology (types of loops [16]) [10, 12, 13]. Sequence restraints may hide rare non-canonical motif variants, such as the U-minor variant of the A-minor interaction motif [17]. Searching by interaction network relies on pairwise interaction annotations that differ significantly depending on the tool used [8]. The topology restraints pose the greatest limitation, resulting in just a few examples of structural similarities identified to date between motifs of different topologies [8, 17–19]. Although tools for topology-independent nucleic acid 3D structure superposition exist, they are designed for the global 3D structure alignment problem and are not suitable for motif search [20–22]. These limitations cause current approaches to favor loop motifs over long-range motifs and substantially hinder the identification of structural similarities between the motifs formed within different backbone topology contexts.

**Fig. 1:**
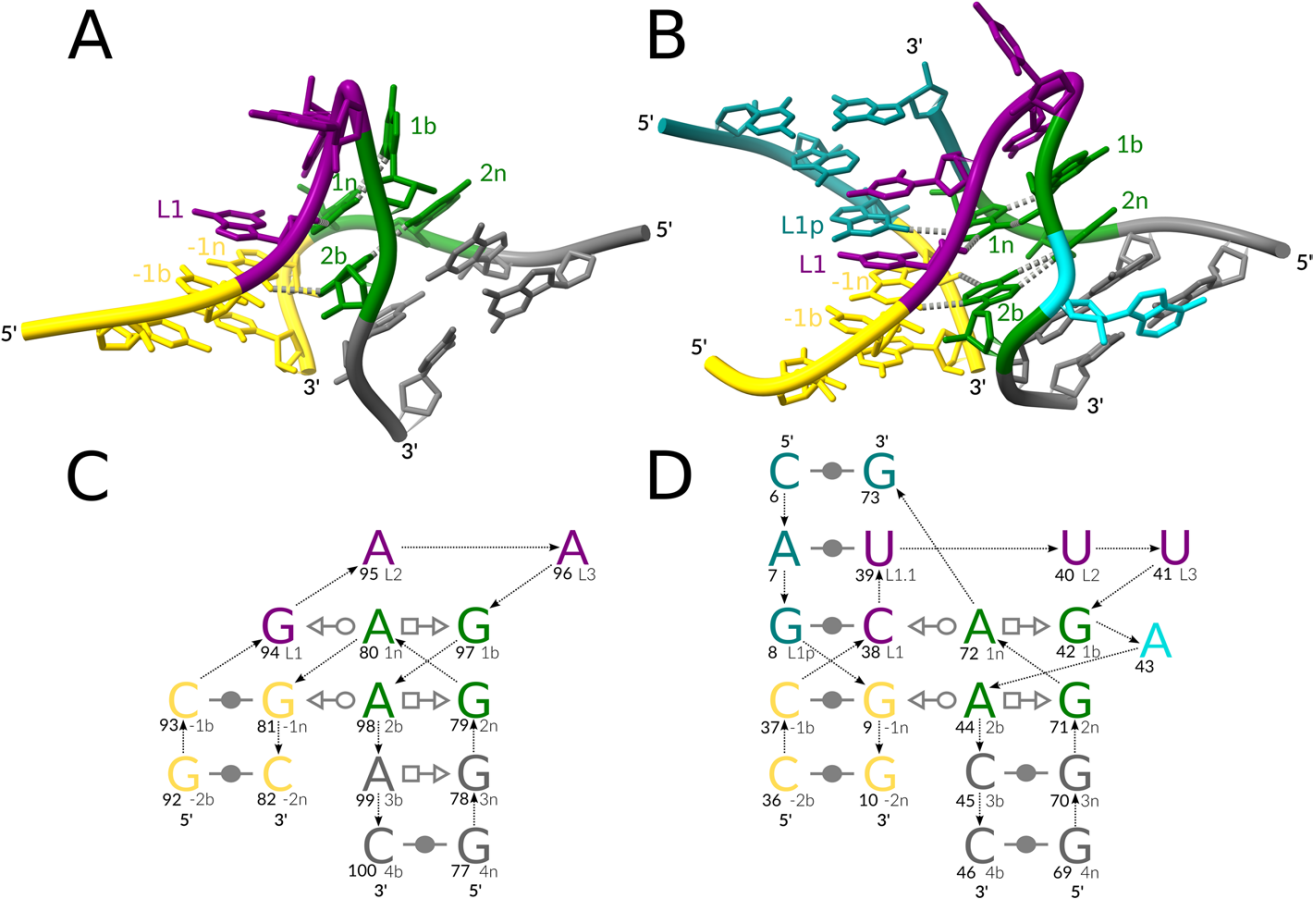
Reference kink-turn motifs. (**A**) A 3D structure of the canonical Kt7 kink-turn [5], LSU rRNA, PDB entry 1FFK, chain 0. (**B**) A 3D structure of the k-junction [5], TPP riboswitch, PDB entry 3D2G, chain A. The interaction schemes of (**C**) the kink-turn and (**D**) the k-junction follow the commonly used nomenclature [5]: the canonical stem (C-stem) in gold, the non-canonical stem (NC-stem) in green and gray, the residues of the kink in purple, the third stem (T-stem) and other stems in teal, and the looped-out residues in dark turquoise. The base pair representation follows the Leontis-Westhof (LW) classification [15]. The backbone connectivity is depicted with arrows. 5'- and 3'-ends are shown accordingly. Each base is marked with its number in the chain (in bold, left) and its named position in the kink-turn (right). The 3D representations were prepared with ChimeraX [29], with key residues labeled and prominent key hydrogen bonds indicated with gray dashed lines. The 2D representations were prepared with Inkscape [30]

The kink-turn motif [23], one of the most well-studied RNA tertiary motifs [5, 24], exemplifies the consequences of the aforementioned limitations. Kink-turns are typically characterized by a canonical stem (C-stem) followed by a bulge that forms a sharp kink in the backbone and a non-canonical stem (NC-stem) starting with *trans-Sugar-Hoogsteen* (*tSH*) *G-A* base pairs [5] (Fig. 1). The kink-turn architecture of an internal loop was discovered in one of the first experimentally resolved ribosomal RNA 3D structures in 2001 [23]. The kink-turn architecture of a three-way junction loop was discovered only in 2011 [18] and later described as the k-junction motif in 2014 [19], despite its multiple instances in the same 3D structures of ribosomal RNAs [19]. It was shown that kink-turns are extremely widespread, with examples in spliceosomal RNAs [25], group I introns [26], and riboswitches [19], playing roles in many aspects of RNA function, such as serving as a protein binding site [27]. Mutations disrupting the kink-turn structure in the spliceosomal U4atac RNA have been shown to cause various diseases, such as Taybi–Linder syndrome [25] and Roifman syndrome [28]. Although the resemblance between kink-turns and diverse A-minor junctions was briefly mentioned as early as 2011 [18], the variety of discovered kink-turns remains limited to the two mentioned topologies [5].

Recently, we developed ARTEM [8], a method for sequentially unconstrained superposition of RNA 3D modules. Here, we present a new version of the ARTEM approach adapted to the tertiary motif search problem. We demonstrate the practical utility of the algorithm to identify motifs structurally similar to the kink-turn motif. First, ARTEM outperformed existing tools in identifying canonical kink-turn motifs. Second, we identified two new kink-turn topologies: a five-way junction and an external loop. We also discovered several kink-turn-like motifs, including A-minor junctions [18] and long-range motifs [8], that preserve the core interactions of a kink-turn but do not involve the kink. Finally, we demonstrate that the 23S rRNA J94/99 junction, previously identified as a k-junction in *H.marismortui* [19], adopts the no-kink junction topology in other bacteria with a slight distortion of the core kink-turn interactions. Additionally, we identified the coordination loop in group II introns as a kink-turn, although group II intron structures have not been previously characterized as containing kink-turns. Furthermore, we used ARTEM to search for four types of RNA and DNA tertiary motifs, demonstrating its suitability for structural motifs of varying nucleic acid types, sizes, interaction networks, and backbone connectivities. ARTEM can be uniformly applied to search for both local and long-range motifs, and its advantages open a fundamentally new way to study nucleic acid 3D folds and motifs, and to analyze their correlations and variations.

## Results

We developed ARTEM 2.0 [31], a new version of the ARTEM algorithm tailored to the nucleic acid tertiary motif search problem. This tool enables automated searches of RNA and DNA structure databases against multiple query modules. ARTEM is equipped with a rich set of user-defined parameters to specify particular 3D structure regions of interest, impose restraints on the identified motifs, and save the superimposed matches or entire nucleic acid-containing input complexes in PDB or mmCIF format. ARTEM advances beyond the limitations of the existing tools by exclusively relying on motif isostericity. It applies to any chosen reference motif, without differentiation between loop motifs and long-range motifs. We exemplified its capabilities by searching for kink-turn-like RNA tertiary motifs. Additionally, we demonstrated ARTEM’s broad applicability by searching for well-known nucleic acid structural motifs, such as G-quadruplex, GNRA tetraloop, and i-motif, as well as a unique RNA motif with four parallel base pairs recently described [32].

### Kink-turn motif identification

To search for instances of the kink-turn motif, we selected the two known backbone topology variants (Fig. 1, Table 1): an instance of the kink-turn internal loop from a 23S rRNA [33] (module #1) and an instance of the k-junction variant from a TPP riboswitch [34] (module #2). We executed ARTEM for the two instances against all RNA-containing entries in the Protein Data Bank [35], see the Methods section for details. The results included 26,564 matches (Additional file 1: Table S1). We annotated the matches with their backbone topology characteristics using urslib2 [8] to facilitate subsequent analysis.

**Table 1:** Selected instances of the kink-turn motif variants

#	PDB entry, chain	Description	Topology	RNA molecule	L1 residue	1n residue	Variant	Figure
1	1FFK chain 0	kink-turn Kt7 reference	internal loop	23S rRNA	G94	A80	kink	1 A, 1C
2	3D2G chain A	k-junction reference	3-way junction	TPP riboswitch	C38	A72	kink	1B, 1D
3	7P7T chain A	J4/5	5-way junction	23S rRNA	G48	A117	kink	3 A, 3B
4	7PKT chain 8	[J99/101]	external loop	L8 rRNA	A453	A481	kink	S1A, S1C
5	7ZHG chain 2	no kink but stem	3-way junction	16S rRNA	G1073	A1032	no-kink	S2A, S2C
6	8EUY chain 1	no kink but two stems	5-way junction	28S rRNA	G973	A685	no-kink	S2B, S2D
7	6ME0 chain A	no kink but stem	4-way junction	group II intron	C141	A181	no-kink	4 A, 4C
8	7PWO chain 1	long-range motif	helix-helix interface	28S rRNA	C1572	G2651	no-kink	4B, 4D
9	4V8O chain BA	J99/101	3-way junction	23S rRNA	G2830	A2883	kink	S1B, S1D
10	3CC2 chain 0	J94/99	complex pseudoknotted junction	23S rRNA	G2823	A2914	kink	5 A, 5C
11	4V8O chain BA	J94/99	complex pseudoknotted junction	23S rRNA	C2788	A2892	no-kink	5B, 5D

**Fig. 2:**
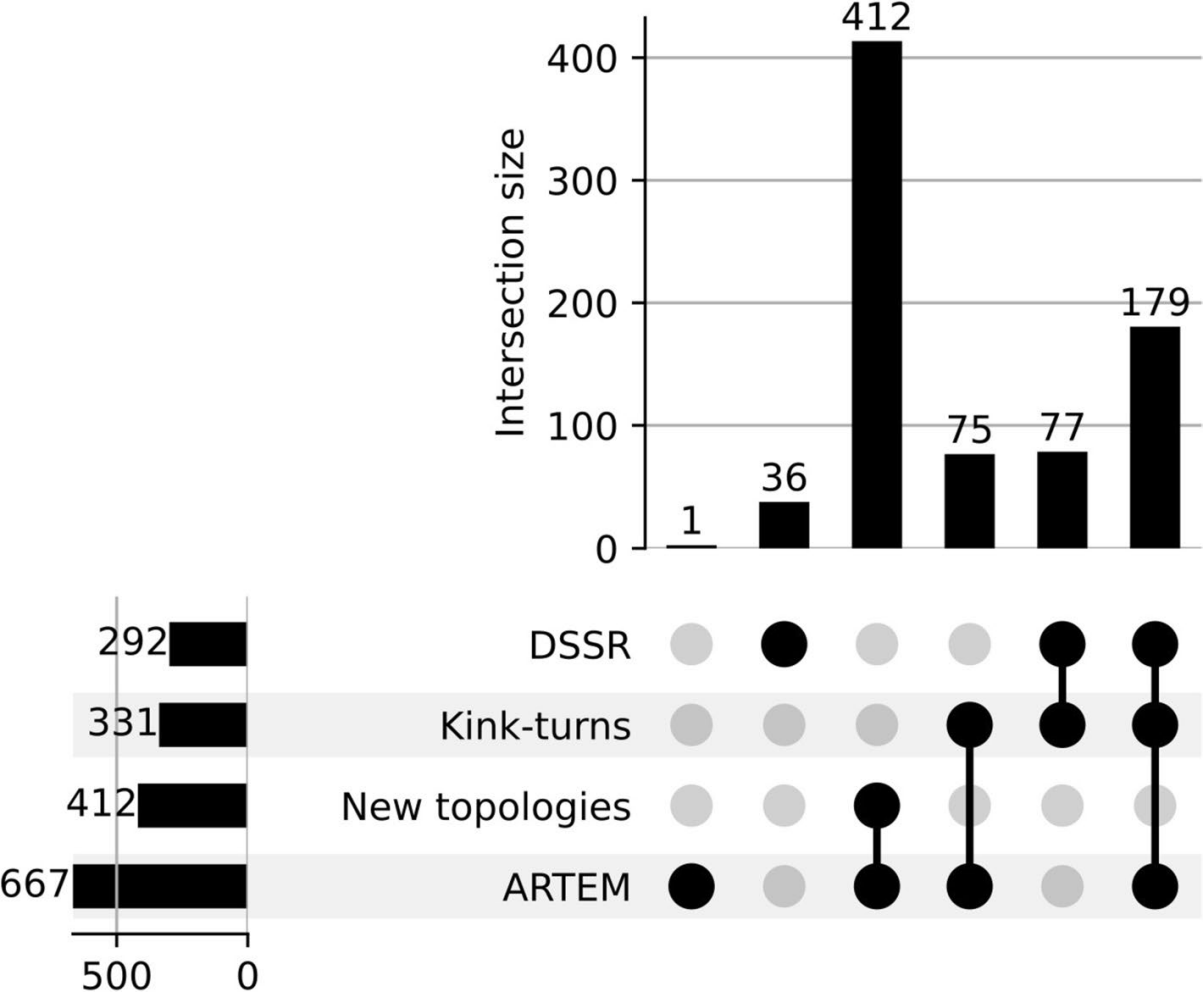
Benchmark of ARTEM and DSSR in identifying kink-turn motifs. The benchmark was performed on 2,394 PDB entries from the BGSU representative set of RNA structures. The UpSet plot was prepared using the UpSetPlot Python library [37]

We benchmarked ARTEM against DSSR [11] using the BGSU representative set of RNA structures [36], which included 2,394 PDB entries (Fig. 2, Additional file 2: Table S2), see the Methods section for details. Within this set, ARTEM identified 255 matches annotated with the canonical internal loop topology, and 412 new topological variants that could not be detected by any other tool. Among the 255 hits, one was confirmed as a false positive: a double-kink internal loop with all canonical base pairs. Although it matched the reference kink-turn at 1.976 Å RMSD, it was manually rejected. In contrast, DSSR identified 292 kink-turns, including 36 false positives. Thus, in identifying canonical kink-turn topologies, ARTEM demonstrated higher precision than DSSR (99.6% vs. 87.7%), while achieving comparable recall (76.7% vs. 77.3%).

We analyzed the false negatives of ARTEM and DSSR and attributed them to limitations inherent to their respective approaches. DSSR searched for internal loops with a specific angle between the stems and a tSH G-A base pair. As a result, it missed true instances featuring alternative base pairs and captured false matches lacking the characteristic arrangement of residues at positions L1, −1b, −1n, 1b, 1n, 2b, and 2n. In contrast, ARTEM searched for isosteric hits of the reference motifs matching at least 12 residues at RMSD ≤ 2.0 Å, and it required positions L1, −1b, −1n, 1b, 1n, 2b, and 2n to have a match. Consequently, ARTEM missed instances lacking some of these required positions (most frequently 1b and 2n), and those that were insufficiently isosteric with the two references.

### New backbone topology variants of the kink-turn motif

**Fig. 3:**
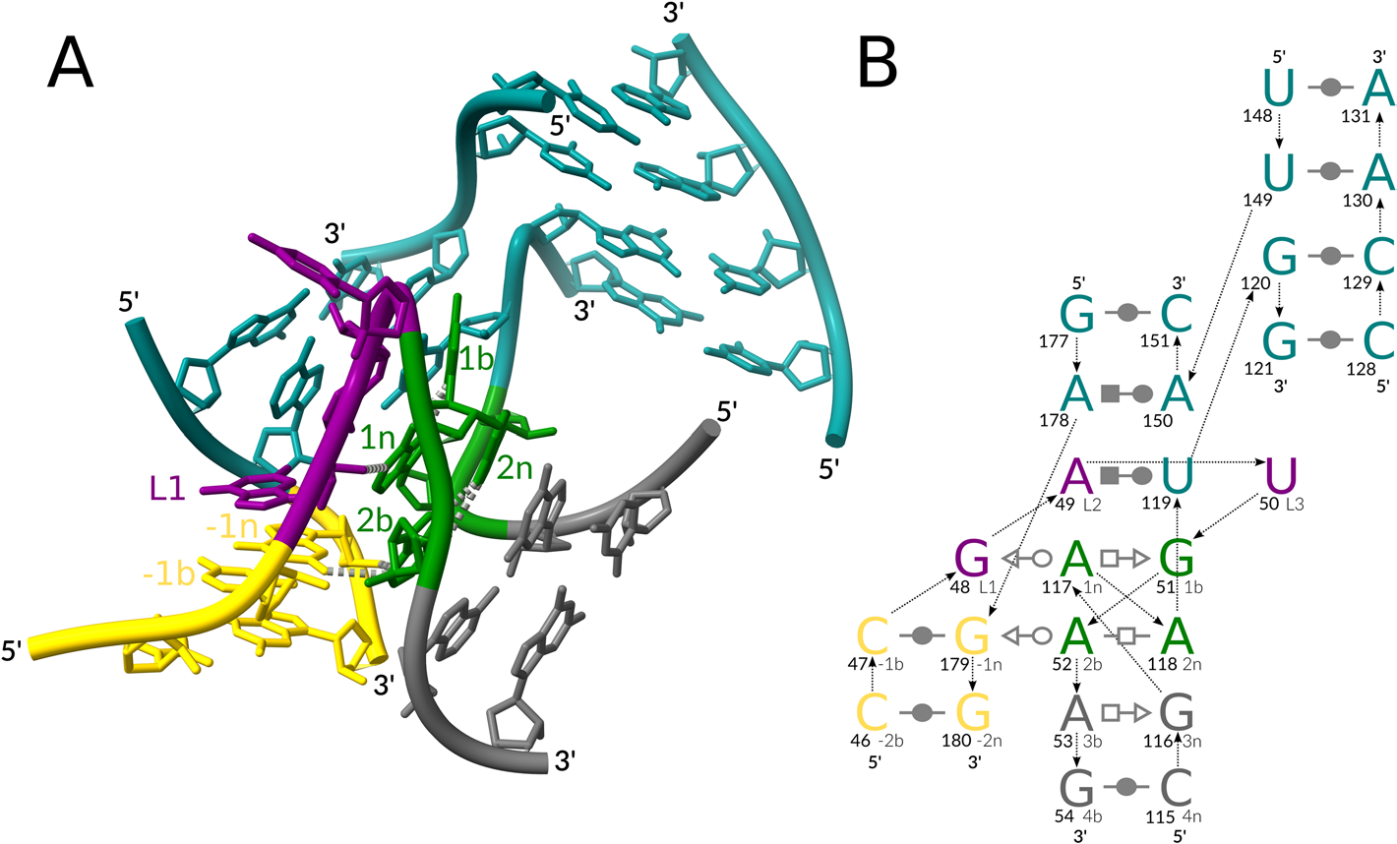
A J4/5 five-way k-junction module. (**A**) A 3D structure of the J4/5 five-way k-junction, LSU rRNA, PDB entry 7P7T, chain A. (**B**) A 2D interaction scheme of the junction: the canonical stem (C-stem) in gold, the non-canonical stem (NC-stem) in green and gray, the residues of the kink in purple, and the other stems in teal. The base pair representation follows the Leontis-Westhof (LW) classification [15]. The backbone connectivity is depicted with arrows. 5′- and 3′-ends are shown accordingly. Each base is marked with its number in the chain (in bold, left) and its named position in the kink-turn (right). The 3D representation was prepared with ChimeraX [29], with key residues labeled and prominent key hydrogen bonds indicated with gray dashed lines. The 2D representation was prepared with Inkscape [30]

Via manual inspection of the ARTEM matches with new topologies, we identified instances of two new variants of the kink-turn motif (Table 1): a five-way junction in a 23S rRNA [38] (module #3, Fig. 3) and an external loop in a mitochondrial LSU rRNA fragment [39] (module #4, Additional file 3: Fig. S1A, C). Below, we use the commonly accepted nomenclature of the kink-turn structure [5], see the Methods section for details.

We mapped the identified five-way junction (module *#3*) to the *J4/5* region of the 23S rRNA structure. The J4/5 region was previously reported to form a k-junction [19], but it was incorrectly identified as a three-way junction. The interaction network of the J4/5 region is closer to the reference module #1, with a *tSH G-A* base pair in position *3n-3b,* but it has a non-linear matching of residues *1n* and *2n,* resulting in a *tHH 2n-2b* base pair.

The external loop (module *#4*) was mapped to the *J99/101* region of the 23S rRNA, which had not been previously reported as a kink-turn or a k-junction. The *J99/101* region forms a canonical three-way k-junction architecture in non-mitochondrial 23S rRNAs (module #9, Additional file 3: Fig. S1B, D). Its interaction network is very close to that of the reference module #2, with one substantial difference: the *2n-2b* base pair is a canonical *cWW* base pair, which is incompatible with the *tSW* interaction formed between residues *−1n* and *2b* in the reference modules.

### No-kink variants of the kink-turn motif

**Fig. 4:**
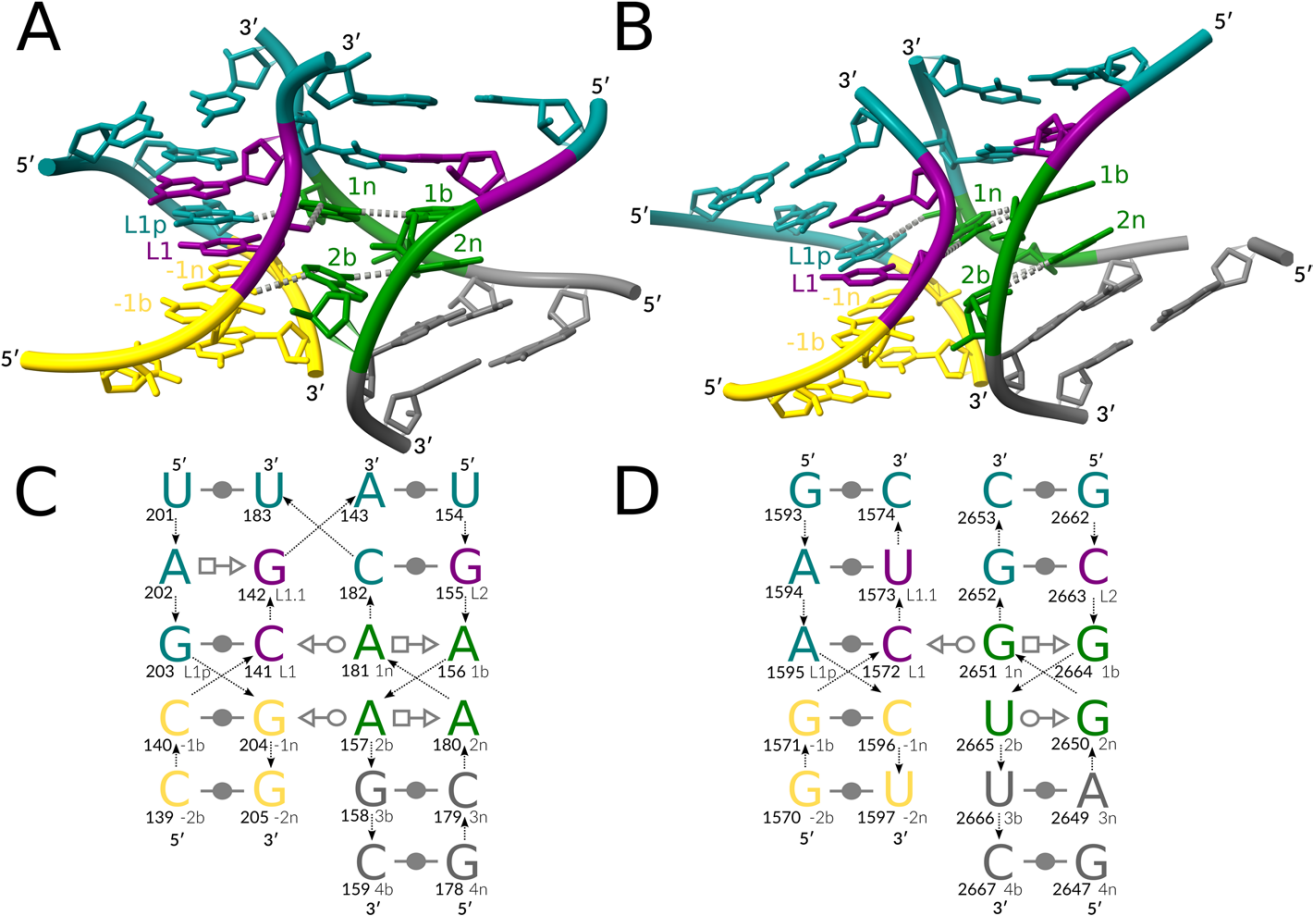
Selected no-kink variants #7 and #8. (**A**) A 3D structure of the four-way A-minor junction, group II intron, PDB entry 6ME0, chain A. (**B**) A 3D structure of the long-range helix-helix interface, 28S rRNA, PDB entry 7PWO, chain 1 (residue 2648 is missing in the coordinate file). The interaction schemes of (**C**) the four-way junction and (**D**) the long-range motif: the canonical stem (C-stem) in gold, the non-canonical stem (NC-stem) in green and gray, the residues matching the kink in purple, and the other residues in teal. The base pair representation follows the Leontis-Westhof (LW) classification [15]. The backbone connectivity is depicted with arrows. 5′- and 3′-ends are shown accordingly. Each base is marked with its number in the chain (in bold, left) and its named position in the kink-turn (right). The 3D representations were prepared with ChimeraX [29], with key residues labeled and prominent key hydrogen bonds indicated with gray dashed lines. The 2D representations were prepared with Inkscape [30]

We observed matches of several unique backbone topologies that lacked the kink while exhibiting the C-stem/NC-stem arrangement isosteric to that of the kink variants (modules #5-#8, Table 1, Fig. 4, Additional file 3: Fig. S2). Three of these modules (#5-#7) were of different junction loop architectures, all of which can be classified as A-minor junctions, whose resemblance to kink-turns was reported previously [18]. In contrast, module #8 is not a loop but a helix-helix interface, showcasing the long-range kink-turn-like motif for the first time.

The no-kink three-way junction (module #5, Additional file 3: Fig. S2A, C) and the no-kink five-way junction (module #6, Additional file 3: Fig. S2B, D) both have a pyrimidine in position *1b* instead of the commonly found guanosine. While this is the only notable difference for module #6, module #5 also features a pyrimidine in position *2n* and a non-linear matching of residues *1b* and *2n,* with *1b* belonging to the non-bulge strand.

The no-kink four-way junction (module #7, Fig. 4A, C) is the closest module to the reference variants in terms of interactions, featuring the same geometric types of the four core base pairs: tSW L1-1n, tSW −1n-2b, tHS 1n-1b, and tHS 2b-2n. The long-range motif (module #8, Fig. 4B, D) is the only match that features a non-adenosine (guanosine) *1n* residue. It also includes a uridine in *syn* conformation at position *2b* that does not interact with the *−1n* residue. Notably, there is a striking 3D structure similarity between modules #7 and #8 (Fig. 4A,B), with ARTEM reporting a 17-residue match at 1.6 Å RMSD between the two modules. Their four RNA strands are arranged similarly but form canonical base pairs with different partners (see the top two base pairs in Fig. 4), resulting in distinct topologies: a four-way junction and a long-range helix-helix interface.

### The identified kink/no-kink switch

**Fig. 5:**
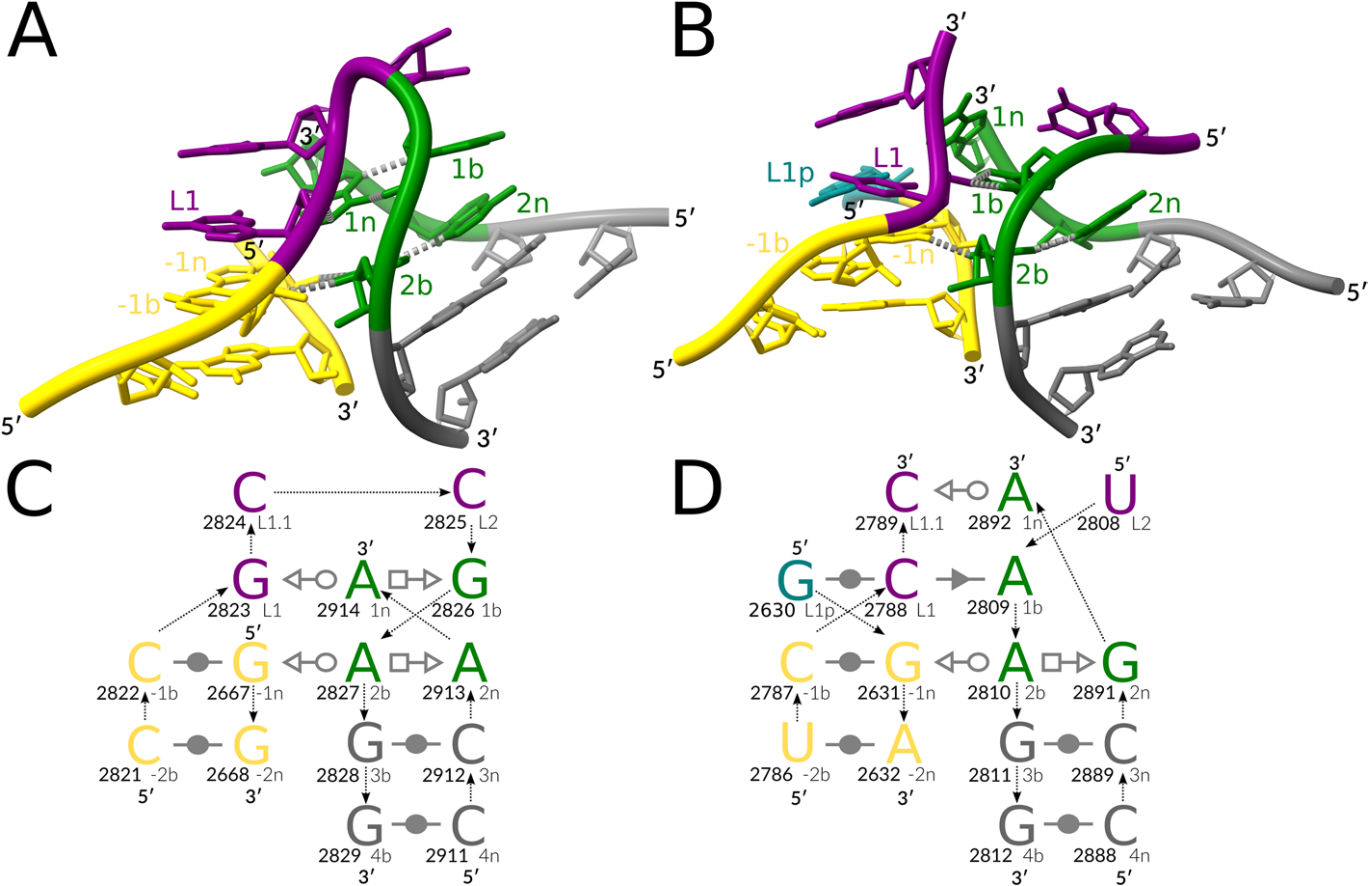
Selected J94/99 region modules. (**A**) A 3D structure of the J94/99 k-junction in H.marismortui, 23S rRNA, PDB entry 3CC2, chain 0. (**B**) A 3D structure of the J94/99 no-kink variant, LSU rRNA, PDB entry 4V8O, chain BA. The interaction schemes of (**C**) the J94/99 k-junction and (**D**) the no-kink variant: the canonical stem (C-stem) in gold, the non-canonical stem (NC-stem) in green and gray, the residues of the kink in purple, and the other residues in teal. The base pair representation follows the Leontis-Westhof (LW) classification [15]. The backbone connectivity is depicted with arrows. 5′- and 3′-ends are shown accordingly. Each base is marked with its number in the chain (in bold, left) and its named position in the kink-turn (right). The 3D representations were prepared with ChimeraX [29], with key residues labeled and prominent key hydrogen bonds indicated with gray dashed lines. The 2D representations were prepared with Inkscape [30]

We discovered that the *J94/99* region of 23S rRNA, another ribosomal k-junction identified previously in *H.marismortui* [19] (module #10, Fig. 5A, C), adopts the no-kink junction topology in other bacteria (module #11, Fig. 5B, D) with a slight distortion of the core kink-turn interactions (Fig. 5D).

In module #11, Helix 98, absent from the *H.marismortui* 23S rRNA [40], is inserted between residues *L1.1* and *L2* that form the kink in module #10. The *1n* adenosine in the no-kink variant is pushed out of its position by residue *1b* and interacts with residue *L1.1* instead of *L1* (Fig. 5D). Also, both modules #10 and #11 formally belong to complex pseudoknotted junction loops, which may be considered yet another backbone topology variant.

### Conservation analysis of ribosomal k-junctions

We analyzed the sequence conservation of the three k-junctions identified in 23S rRNAs (Additional file 3: Fig. S3). The conservation patterns differ substantially between the junction regions, with no conserved positions (defined as having an information content > 1.5 bits for a single base) shared among all three motifs. The J4/5 pattern shares three conserved positions with J94/99 (−1b = C, −1n = G, and 2b = A) and four other conserved positions with J99/101 (1n = A, 1b = G, 2n = A, and 3b = A), while the sets of conserved positions in J94/99 and J99/101 show no overlap.

The *J4/5* region exhibits the largest number of conserved residues (10), including the *tHS A-G* base pair in position *3b-3n,* which is also characteristic of the canonical Kt7 kink-turn (module #1). The *1n* residue in the *J94/99* region is the least conserved *1n* residue among the junctions, consistent with its distorted position in the presence of *Helix 98* (module #11). Surprisingly, the *2b-2n cWW U-A* base pair is highly conserved in the *J99/101* region, while the *J99/101* C-stem base pairs are the least conserved among the junctions.

### The coordination loop in group II introns is a kink-turn

**Fig. 6:**
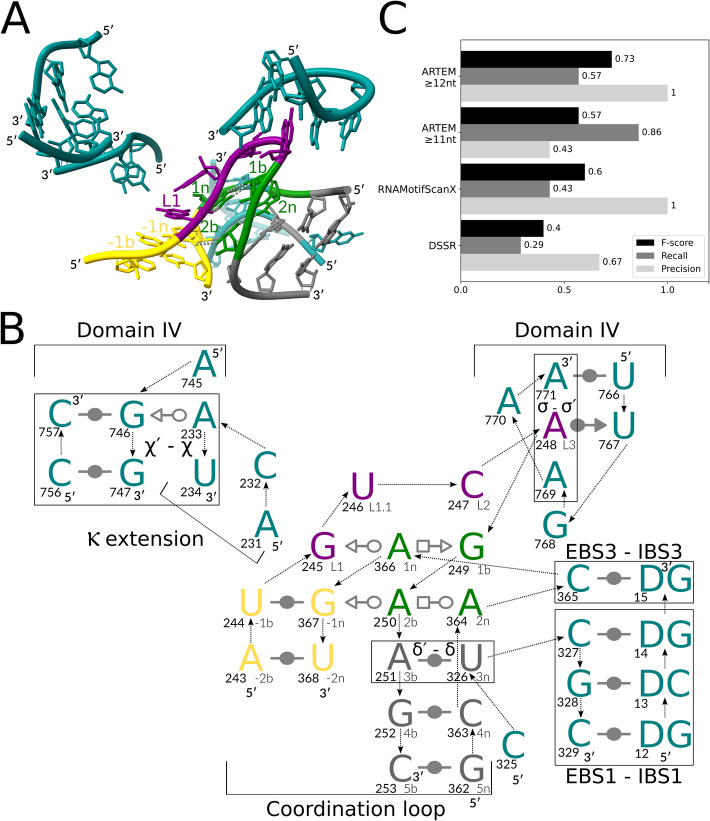
Group IIC intron coordination loop. (**A**) A 3D structure of the coordination loop kink-turn, group IIC intron, PDB entry 6ME0, chain A. (**B**) The interaction scheme of the kink-turn: the canonical stem (C-stem) in gold, the non-canonical stem (NC-stem) in green and gray, the residues of the kink in purple, and the other residues in teal. The base pair representation follows the Leontis-Westhof (LW) classification [15]. The backbone connectivity is depicted with arrows. 5′- and 3′-ends are shown accordingly. Each base is marked with its number in the chain (in bold, left) and its named position in the kink-turn (right). The tertiary interactions (Greek letters) and the intron regions are marked according to the conventional naming [41, 42]. (**C**) ARTEM benchmarking against other tools that identified at least one of the seven kink-turns in the seven representative group II intron structures. The 3D representation was prepared with ChimeraX [29], with key residues labeled and prominent key hydrogen bonds indicated with gray dashed lines. The 2D representation was prepared with Inkscape [30]. The barchart was prepared with Matplotlib [43]

While manually inspecting the kink-turn matches obtained with ARTEM, we identified a kink-turn of the canonical internal loop architecture in a 3D structure of group IIC intron [41] (Fig. 6A). To the best of our knowledge, occurrences of kink-turns in group II intron structures were never previously reported. To close this gap, we selected a set of seven group II introns from the representative set of RNA structures [36] and surveyed them for kink-turns, both manually and using ARTEM, DSSR [11], and RNAMotifScanX [14], see the Methods section for details.

We identified seven kink-turn modules in the seven group II intron structures (Additional file 3: Table S3). Five of the seven kink-turn modules were found in the functional intron region of domain I (DI) known as the coordination loop [42], present in the structures of group IIB and group IIC introns. As implied by its name, the loop coordinates the formation of the intron’s catalytic core by interacting with the Κ extension bulge and the exon binding site 1 (EBS1) loop [42]. Additionally, the coordination loop encompasses the single-residue exon binding site 3 (EBS3). Subsequently, exon–intron recognition is facilitated through interactions with the intron binding sites IBS1 and IBS3 (Fig. 6B). The remaining two kink-turns were identified as instances of a single Domain III (DIII) kink-turn module in group IIC introns.

The kink-turn architecture is crucial for the functioning of the coordination loop (Additional file 3: Table S3). The residues *L1, L1.1*, and *L2* interact with the Κ extension. The *L3* residue forms a base wedged element (BWE) [44] with a Domain IV (*DIV*) loop prior to DNA integration [41]. In the group IIC intron structures, the residues *L2, L3, 1b*, and *EBS3* are exposed to the reverse transcriptase protein. Furthermore, the unusual long-range δ′-δ base pair in position *3b-3n* facilitates the stacking between *EBS3* and *EBS1*. Notably, the two representative group *IIA* intron structures lack *EBS3*, and their coordination loops do not form a kink-turn.

Surprisingly, the coordination loop was never identified as a kink-turn previously. Possibly, the long-range δ′-δ base pair replacing the local *3b-3n* base pair and the looped-out *EBS3* residue hid the motif from identification via sequence- and backbone-restrained kink-turn searches. DSSR failed to identify any of the coordination loop kink-turns and found only the two DIII kink-turn modules. RNAMotifScanX, searching with the consensus kink-turn interaction network, identified the two DIII kink-turns and a single coordination loop kink-turn. In contrast, ARTEM, searching with a single Kt7 reference module, identified four of the five coordination loop kink-turns with the 12nt threshold on the match size. With the 11nt threshold, ARTEM identified all five coordination loop kink-turns and one of the DIII kink-turns, along with eight false positive matches (matches of non-canonical backbone topologies). Consequently, ARTEM outperformed the existing tools, demonstrating an impressive recall of 57% at 100% precision with the 12nt threshold, and 86% recall at 43% precision with the 11nt threshold (Fig. 6C, Additional file 4: Table S4).

### ARTEM can detect various types of RNA and DNA tertiary motifs

To demonstrate the broad applicability of ARTEM, we also used it to search for four types of nucleic acid tertiary motifs across all RNA- and DNA-containing PDB entries. This search against complete but redundant datasets aimed to verify the characteristic features of the motifs and to define optimal sequence and RMSD restraints for ARTEM use. Three of the four motifs are well-known: the parallel G-tetrad [45] (Additional file 3: Fig. S4A), GNRA tetraloop [46] (Additional file 3: Fig. S5), and i-motif [47] (Additional file 3: Fig. S6). The fourth is an unusual motif of four base pairs formed between parallel-oriented strands (Additional file 3: Fig. S7), recently identified in place of a predicted pseudoknot in the crystal structure of cap-independent translation enhancers (CITE) from Pea enation mosaic virus RNA 2 (PEMV2) [32]. We refer to this motif as the parallel-pairing motif.

Distinct RMSD distributions of all-guanine and non-all-guanine matches (Additional file 3: Fig. S4B, C) confirmed the high specificity of the parallel G-tetrad motif to guanosine bases, with only a few non-guanine matches identified with RMSD < 1.0 Å, such as an all-adenine tetrad (Additional file 3: Fig. S4D). In contrast, at RMSD from 1.0 to ~ 1.75 Å, we observed a notable portion of false all-guanine matches that spanned residues from several adjacent non-parallel tetrads (Additional file 3: Fig. S4E, F). Therefore, an RMSD threshold of 1.0 Å is recommended when searching for parallel tetrads with ARTEM, with additional sequence restraints if necessary. Overall, ARTEM identified tetrads with RMSD under 1.0 Å in 418 PDB entries, 417 of which are registered in the DSSR-G4DB database [48] as containing G-quadruplexes, while the remaining entry (PDB 5M1L [49]) involves non-canonical GAGA-tetrads.

Unexpectedly, among GNRA tetraloop matches identified at RMSD < 1.0 Å, we did not observe clear separation between RMSD distributions of GNRA and non-GNRA matches (Additional file 3: Fig. S5B). Among these matches, 6.8% were GAAG tetraloops, and 4.7% were UAAC tetraloops, which adopted the same fold but did not conform to the GNRA pattern. To account for redundancy, we analyzed non-coding RNA families containing these matches. The GAAG loop was found exclusively in ribosomal RNAs (both SSU and LSU, across seven RNA families) and was further stabilized either by RNA–protein interactions (LSU) or by a base-phosphate interaction (SSU, see Additional file 3: Fig. S5D). The UAAC loop was identified only in a lariat capping ribozyme and in bacterial SSU rRNA, where it was stabilized by a distant adenosine base in both cases (Additional file 3: Fig. S5E). As expected, non-GNRA loops require additional stabilization to form a GNRA-like fold. Interestingly, ARTEM identified a single GNRA tetraloop match formed by a DNA chain, indicating that this motif is not exclusive to RNA (Additional file 3: Fig. S5C). Overall, an RMSD threshold of 1.5 Å is recommended when searching for GNRA tetraloops with ARTEM, as matches with distorted folds appear above this threshold (Additional file 3: Fig. S5F). Out of 1,304 GNRA tetraloop matches identified by ARTEM at RMSD under 1.5 Å in the BGSU representative set, 1,140 (87.4%) were also identified by DSSR [11] or rna_motif utility of Rosetta package [50], while the remaining 164 matches were manually confirmed to adopt a GNRA-like conformation, with some hydrogen bonds exceeding the common thresholds (Additional file 3: Fig. S8).

Among RNA-containing PDB entries, ARTEM identified only 12 matches for the reference RNA i-motif instance at RMSD < 2.0 Å, all being all-cytidine matches at RMSD < 0.35 Å from the same PDB entry as the reference (Additional file 3: Fig. S6C). Thus, PDB entry 1I9K is the only entry containing an RNA i-motif, confirming that i-motifs are generally unfavorable in RNA structures. Among DNA-containing PDB entries, ARTEM identified 144 all-cytidine i-motif matches at RMSD < 1.0 Å, one match involving thymine-thymine base pairs at RMSD = 1.004 Å, and four matches involving adenosine-adenosine base pairs at RMSD = 1.24 Å (Additional file 3: Fig. S6B, D, E). Overall, ARTEM identified i-motif matches with RMSD under 2.0 Å in 18 PDB entries, 17 of which were also annotated as i-motif-containing by DSSR, while the remaining entry (PDB 1C11 [51]) was flagged by DSSR as containing a 10nt segment of an i-motif, below the tool’s 12nt size threshold.

Across RNA- and DNA-containing PDB entries, ARTEM did not identify a single match for the parallel-pairing motif at RMSD < 1.0 Å. The closest match was found at RMSD = 1.679 Å and did not exhibit the parallel-pairing pattern characteristic of the reference motif (Additional file 3: Fig. S7D, E, F). Therefore, this search confirms the uniqueness of the parallel-pairing motif described in the CITE RNA from PEMV2 [32]. Additionally, we searched for the PEMV2 parallel-pairing motif in the saguaro cactus virus (SCV) CITE RNA structure, released after we constructed our search datasets (PDB entry 8T29 [52]). In this structure, the motif lacks one residue, causing ARTEM to identify only a seven-residue match at 1.19 Å RMSD (Additional file 3: Fig. S7G, H, I). These analyses demonstrate ARTEM’s unique suitability for identifying similarities between nucleic acid structural motifs, regardless of the nucleic acid type, size, interaction network, or backbone connectivity.

## Discussion

In this work, we developed ARTEM 2.0, an adaptation of the ARTEM algorithm suitable for tackling the long-standing problem of RNA tertiary motif search. ARTEM relies exclusively on motif isostericity and enables the identification of topology-independent structural similarities between the motifs. Therefore, ARTEM overcomes the “*clear shortcoming in classifying RNA motifs simply based on their secondary structures*” [18], as it is capable of finding similar 3D residue arrangements formed within different contexts of secondary structure elements, i.e., stems, loops, and strands. This method applies to any chosen RNA or DNA 3D module, without differentiation between loop motifs and long-range motifs.

We employed ARTEM to explore the landscape of modules isosteric to the kink-turn motif. ARTEM outperformed existing tools in identifying kink-turns with canonical internal loop topology and uncovered new topological variants, including a five-way junction and an external loop. We showed that certain motifs preserve the same core interactions of the kink-turn but lack the kink. We demonstrated that the ribosomal region *J94/99* can form either a kink or a no-kink variant of the motif depending on the species, albeit with a distortion in residue arrangement in the no-kink variant. Furthermore, we identified kink-turns with a complex network of long-range base pairs in the catalytic core of group II introns, whose structures were never previously reported to involve kink-turns. Analyzing the identified kink-turn modules, we found that their only consensus feature is the *1n* purine (almost always adenosine) forming the type I A-minor interaction [53] with the residue *L1* (and its partner *L1p* if present) and donating its Hoogsten edge for base-pairing with the residue *1b*. Strikingly, a 3D module can even lack this essential feature and still be largely isosteric to kink-turns, as evidenced by module #11 (Fig. 5B,D). Therefore, in our view, the minimal structural characteristics required to define a kink-turn-like motif are the “turn” formed between the two stems and the 1n-L1 type I A-minor at the core of this turn, with the “kink” feature serving as the criterion to distinguish kink-turn and kink-turn-like motifs.

Additionally, we used ARTEM to search for four distinct types of nucleic acid structural motifs: parallel G-tetrad, GNRA tetraloop, i-motif, and parallel-pairing motif. For example, we demonstrated that GNRA-like folds can be formed by non-GNRA loops with additional stabilization provided by distant residues or other molecules (Additional file 3: Fig. S5). Benchmarking of ARTEM against DSSR and rna_motif confirmed its high precision across G-tetrads, GNRA tetraloops, and i-motifs. Thus, we showed that ARTEM is readily applicable to structural motifs of various sizes, interaction networks, and backbone connectivities.

A notable limitation of ARTEM is that it may require some fine-tuning based on the particular motif of interest and the desired *FP/FN* ratio. In turn, ARTEM offers high flexibility and enables a rich set of user-defined restraints. Nevertheless, we intend to develop a separate tool, tailored for the annotation of the most prevalent RNA and DNA tertiary motifs, that can be used by non-experts as a closed user-friendly solution.

ARTEM introduces a fundamentally new approach to investigating nucleic acid 3D folds and motifs and analyzing their correlations and variations. It extends the concept of isostericity from base pairs [54] to more complex tertiary motifs. By employing a purely isosteric-based tertiary motif search, ARTEM promises to unveil numerous hidden similarities among recurrent RNA and DNA 3D modules, significantly advancing our understanding of the principles governing RNA and DNA 3D structure organization.

## Conclusions

In this work, we present ARTEM 2.0, a computational tool that enables isostericity-based searches for 3D motifs in nucleic acid structure databases and can be uniformly applied to motifs of arbitrary size, interaction network, and backbone topology, without constraining the search by any of these features. We applied ARTEM to search for kink-turns, G-quadruplexes, GNRA tetraloops, and i-motifs. ARTEM outperformed existing methods in identifying kink-turns with canonical internal loop topology and led to the discovery of several new topological variants of kink-turns, as well as no-kink variants of the motif. Furthermore, ARTEM identified an unusual kink-turn with long-range base pairs in the coordination loop of group II introns, whose structures had not previously been reported to feature kink-turns. ARTEM sets a conceptually new approach for identifying nucleic acid 3D folds and motifs and is expected to facilitate the discovery of numerous previously unrecognized recurrent RNA and DNA 3D modules.

## Methods

### Definitions

Below we provide definitions as applied to RNA 3D structures; however, these definitions apply equally to DNA 3D structures. The definitions of RNA secondary structure elements used in this work follow our previous study [8]. A *stem* is a helical region formed by at least two consecutive stacked base pairs, of Watson–Crick G-C/A-U or wobble G-U type, and a *loop* is a set of single-stranded regions confined by a stem. A loop and a stem are called *adjacent* if at least one residue of the loop is a direct neighbor in sequence of a residue of the stem. Two elements (stems or loops) are called *distant* if they are not adjacent and don’t share common adjacent elements. An *internal loop* includes two strands and is adjacent to two stems. An *N-way junction loop* involves *N* > *2* strands and is adjacent to *N* stems. A loop of any type is called *external* if it includes dangling ends among its strands.

An *RNA 3D module* is loosely defined as a set of interacting residues that can be represented with a connected graph. A module is called *long-range* if it involves at least one pair of residues that belong to distant RNA secondary structure elements. Otherwise, a module is called *local*. An *RNA tertiary motif* is defined as a recurrent RNA 3D module. A module is called a *motif instance* if it’s identified as the motif representative. We defined the computational problem of RNA tertiary motif search as the task of identifying instances of a given motif in a query RNA 3D structure having an instance of the motif as the reference.

In this work, we adhere to the established nomenclature of the kink-turn structure [5] and Leontis-Westhof base pair classification [15] (Fig. 1). A residue forms a base pair with another residue via one of its three edges: *Watson–Crick* (*W*, circle), *Hoogsteen* (*H*, square), or *Sugar* (*S*, triangle), in either *cis* (filled) or *trans* (empty) orientation. A canonical kink-turn comprises two helical regions, a canonical stem (C-stem, in gold) and a non-canonical stem (NC-stem, in green and gray), connected by an internal loop. The longer strand of the loop (bulge, in purple) involves the sharp kink in its backbone. The NC-stem starts with two characteristic *trans-Sugar-Hoogsteen* (*tSH*) *G-A* base pairs (in green). The residues of the bulge are labeled as *L1*, *L2*, and *L3*. The base pairs of NC-stem are labeled with positive numbers starting from the base pair adjacent to the bulge, with lowercase *b* (bulge side) or *n* (non-bulge side) letters: *1n/1b* base pair, *2n/2b* base pair, and so on. The C-stem base pairs are labeled with negative numbers: *−1b/−1n* base pair, *−2b/−2n*, and so on. The k-junction variant (Fig. 1B, D) involves an additional *L1.1* bulge residue and the third stem (T-stem, in teal). The *L1* residue of the k-junction forms a canonical base pair with the residue that we labeled *L1p*.

We should note that the NC-stem, as defined in [5], deviates from the strict stem definition, with the *tSH G-A* base pairs formally falling within the internal loop. To maintain consistency, we adhere to the conventional NC-stem definition, while employing the strict stem definition for RNA secondary structure annotation.

### ARTEM tool improvement

The ARTEM algorithm superimposes two input nucleic acid structures of N and M residues using single-residue seeds [8]. For each of the N × M possible superpositions, ARTEM matches mutually closest residues within a given distance threshold and reports the match size and RMSD if the criteria are met. Thus, ARTEM addresses the generally NP-hard problem of matching two arbitrary residue sets in polynomial time, leveraging the ubiquitous planar interactions characteristic of nucleic acid structures. For seed superpositions, ARTEM uses 5-atom residue representations (three base atoms, one ribose atom, one phosphate atom), and for superimposing matches and calculating RMSD, it employs 3-atom base representations.

As the original ARTEM tool [8] reports all local structural similarities identified between the two nucleic acid 3D modules, we modified it to make it more suitable for the nucleic acid tertiary motif search. ARTEM 2.0 introduces three new features compared to its predecessor [31]. First, the input structures (both reference and query) can be specified with a folder or mask instead of a single file, allowing one to search against a set of RNA/DNA structures with one command. Second, we added a *nosub* option to remove sub-matches (matches involved in larger matches), allowing one to filter out partial hits. Third, we introduced an *rst* option, enabling users to specify additional restraints of four different types: (i) filtering out matches missing specified residues, (ii) requiring the specified residues to match with particular base types (allowing the IUPAC nomenclature [55]), (iii) requiring the specified residues to match under a given RMSD threshold, and (iv) requiring the specified residues to match with a continuous nucleic acid strand. The continuity of a strand is verified by O3′-P atom distances under 2.0 Å between neighboring residues.

### Kink-turn motif search with ARTEM

To conduct the kink-turn motif search with ARTEM, we selected one instance for each of the two known backbone topology variants (Fig. 1, Table 1): the canonical kink-turn internal loop (LSU rRNA, PDB entry 1FFK [33], chain 0, *L1* residue *G94*) and the k-junction variant (TPP riboswitch, PDB entry 3D2G [34], chain A, *L1* residue *C38*). For the search, we confined the motif to the following 14 residues: *L1, L1p, −1b, −1n, −2b, −2n, 1b, 1n, 2b, 2n, 3b, 3n, 4b, 4n*. Other labeled residues were excluded due to their dynamic positioning between the kink-turn instances.

To determine the thresholds for the search, we executed ARTEM using the two reference instances as input. ARTEM identified a correct (consistent with labels) match of 13 residues (*L1p* is absent in the canonical kink-turn) at *RMSD* of *1.837 Å* between the two instances. Accordingly, we set the search for the matches of at least 12 residues (*sizemin* = *12*) at RMSD under 2.0 Å (*rmsdmax* = *2.0*). Additionally, we imposed a restraint requiring the matches to include the counterparts of all of the following residues: *L1, −1b, −1n, 1b, 1n, 2b, 2n*, as they are deemed most essential for the kink-turn motif. Preliminary tests showed that lowering the sizemin threshold for the selected references leads to false single-stem matches, while reducing the references to fewer core residues results in false matches lacking the characteristic “turn” arrangement between the two stems.

We then executed ARTEM for each of the two references against all RNA-containing structures in the Protein Data Bank [35] (7,292 PDB entries as of December 7th, 2023). The search concluded within 24 h on an AMD Ryzen 9 5950X machine equipped with 32 CPU cores and 128 GB RAM. ARTEM identified 11,025 matches for the kink-turn and 18,727 matches for the k-junction. Combining the matches identified against both references resulted in 26,564 unique matches (Additional file 1: Table S1). Finally, to facilitate subsequent analysis, we used the *urslib2* Python library [8] to annotate RNA secondary structure elements and additional characteristics (e.g., local/long-range, kink/no-kink) of the hits’ residues based on the base pair annotations obtained with DSSR [11] (version 2.0). The kink/no-kink distinction was approximated by the distance in RNA sequence between the *2b* and *L1* residues: the kink label was assigned to the matches with a distance under ten residues.

Additionally, we ran a similar ARTEM search against all DNA-containing PDB entries (10,505 entries as of December 7th, 2023). We confirmed that no kink-turn or k-junction matches were identified within DNA molecules.

### Analysis of the kink-turn motif search results

We manually inspected the motif matches identified with ARTEM, targeting novel backbone topologies, as annotated with *urslib2*. The analysis was performed using ChimeraX [29]. The boundaries of the identified modules were defined manually, and the modules were then saved as separate coordinate files [56].

The naming of the ribosomal k-junctions *J4/5* and *J94/99* followed the original k-junction paper [19]. The name for the *J99/101* k-junction was derived analogously using the RibosomeGallery [40]. The mapping of the identified motifs to the named ribosomal regions was done based on the original structure papers [38, 39, 57]. We should note that modules #9 (PDB entry 4V8O, *J99/101*) and #10 (PDB entry 3CC2, *J94/99*) were missing from the original list of ARTEM matches (Additional file 1: Table S1). They could be identified with ARTEM only with relaxed thresholds: 11-residue hit at *RMSD* = *1.975 Å* against module #2 and 12-residue hit at *RMSD* = *1.958 Å* missing *−1n* counterpart against module #1, respectively.

For the conservation analysis, we mapped the three k-junctions to the Rfam seed alignment of the 23S rRNA family RF02541 [58]. The relevant sequence regions were then excised from the alignment and used as input for the WebLogo application [59] to construct the motif logos.

### Benchmarking and the group II intron survey for kink-turns

For benchmarking, we downloaded 2,394 PDB entries from the BGSU representative set of RNA structures [36] (version 3.312 with the resolution cutoff of 4.0 Å). For DSSR [11] (version 2.0), we derived the kink-turn annotations with the “Normal kink-turn” label. For RNAMotifScanX [14] (release_v0.0.5) we converted mmCIF files into the PDB format and utilized the built-in kink-turn consensus structure (models/k-turn_consensus.struct). RNAMotifScanX processed only 702 PDB entries and crashed on the remainder. Among the processed entries, RNAMotifScanX identified 11 kink-turns, all of which were also detected by ARTEM and/or DSSR. Consequently, we excluded RNAMotifScanX from the benchmark, limiting the comparison to ARTEM and DSSR. Matches identified by both ARTEM and DSSR were automatically assigned as true positives. Matches identified by only one tool were manually inspected to determine true or false positives (Additional file 2: Table S2). The quality metrics were calculated as follows: precision = TP/(TP + FP), recall = TP/(TP + FN), and F-score = 2*TP/(2*TP + FP + FN).

For the analysis of group II introns, we selected representative RNA structures assigned with the “group II” keyword in the BGSU set [36] (version 3.312 with the resolution cutoff of 4.0 Å). We excluded the 70-nt structure (PDB entry 1KXK) as it is a short intron fragment. The remaining set included seven group II intron structures (Additional file 3: Table S3). We then manually identified seven kink-turn 3D modules in the seven structures: five coordination loops and two DIII kink-turns, all of them with the internal loop architecture.

Overall, we utilized seven different RNA tertiary motif search tools (Additional file 4: Table S4). Although direct comparisons between the tools are often unfeasible, we approached the analysis from the final user perspective, where we have a reference motif instance and a query RNA structure, aiming to identify motif instances within the structure. The 13 residues (*L1, −1b, −1n, −2b, −2n, 1b, 1n, 2b, 2n, 3b, 3n, 4b, 4n*) of the Kt7 kink-turn module (PDB entry *1FFK*, chain *0*, *L1* residue *G94*) were used as the reference motif instance. For all the tools, we considered the identified match to be a true positive (*TP*) if it overlapped with the correct kink-turn by at least 50% of its residues; otherwise, it was classified as a false positive (*FP*). A correct kink-turn instance not identified by the tool was labeled as a false negative (*FN*). Different matches of the same module were merged together before tallying the *TP, FP*, and *FN* values. Four of the tools failed to identify any kink-turns and were excluded from the comparison: WebFR3D [9], JAR3D [10], LocalSTAR3D [12], and CircularSTAR3D [13]. ARTEM was run with no RMSD threshold but with two different minimum match-size thresholds: 12 residues or 11 residues. DSSR and RNAMotifScanX were run similarly to the main benchmark.

### Analysis of other types of RNA and DNA tertiary motifs

Reference instances of the parallel G-tetrad, GNRA tetraloop, i-motif, and parallel-pairing motif were manually selected and searched against all RNA- and DNA-containing PDB entries (7,292 and 10,505 entries, respectively, as of December 7th, 2023) using ARTEM (see REPRODUCE.md [56]). The following thresholds were set for these searches: for the parallel G-tetrad, a minimum match size of 4 residues and a maximum RMSD of 2.0 Å; for the GNRA tetraloop, a minimum match size of 6 residues (tetraloop plus flanking base pair) with the match required to form a single continuous strand; for the i-motif, a minimum match size of 8 residues; and for the parallel-pairing motif, a minimum match size of 8 residues. Each search took approximately 4–8 h on the same machine, with the time nearly linearly proportional to the motif size.

For GNRA tetraloop benchmarking, we used the same BGSU representative set of 2,394 PDB entries as for kink-turns. ARTEM matches were compared to U-turns identified by DSSR and GNRA_TETRALOOP motifs identified by rna_motif from Rosetta (version 3.14, [50]). Since hits reported by DSSR and rna_motif included non-tetraloop instances (e.g., pentaloops with bulged-out bases), whereas ARTEM was limited to single-strand six-residue matches, we considered only ARTEM’s precision and not recall in this benchmark.

## Supplementary Information

Additional file 1: Table S1. 26,564 kink-turn matches obtained with ARTEM.

Additional file 2: Table S2. 780 kink-turn matches used for benchmarking ARTEM against DSSR and RNAMotifScanX.

Additional file 3: Supplementary materials. Fig. S1-S8 and Table S3.

Additional file 4: Table S4. Benchmarking data for group II introns.

## Data Availability

To perform this work we used nucleic acid-containing entries downloaded from the RCSB PDB [60] and the BGSU representative set of RNA structures, version 3.312 [61]. ARTEM 2.0 is freely available at GitHub (https://github.com/david-bogdan-r/ARTEM) [31] and Zenodo (http://doi.org/10.5281/zenodo.14034347) [62] under Apache-2.0 license. The datasets generated during the current study are available at GitHub (https://github.com/febos/ARTEM-KT) [56] and Zenodo (https://doi.org/10.5281/zenodo.11406084) [63].
