# Supplementary material for: ARTEM: a method for RNA and DNA tertiary motif identification with backbone permutations
Source: Genome Biol. 2025 Jul 28;26:226. doi: 10.1186/s13059-025-03696-2 (PMC12306022; doi:10.1186/s13059-025-03696-2)
Supplement: Supplementary file 3 — Additional file 3: Supplementary materials. Fig. S1-S8 and Table S3. [file 13059_2025_3696_MOESM3_ESM.pdf]

Supplementary Materials

Table of Contents:

Fig S1 (included in the PDF)

Fig S2 (included in the PDF)

Fig S3 (included in the PDF)

Fig S4 (included in the PDF)

Fig S5 (included in the PDF)

Fig S6 (included in the PDF)

Fig S7 (included in the PDF)

Fig S8 (included in the PDF)

Table S1 (external XLSX file)

Table S2 (external XLSX file)

Table S3 (included in the PDF)

Table S4 (external XLSX file)

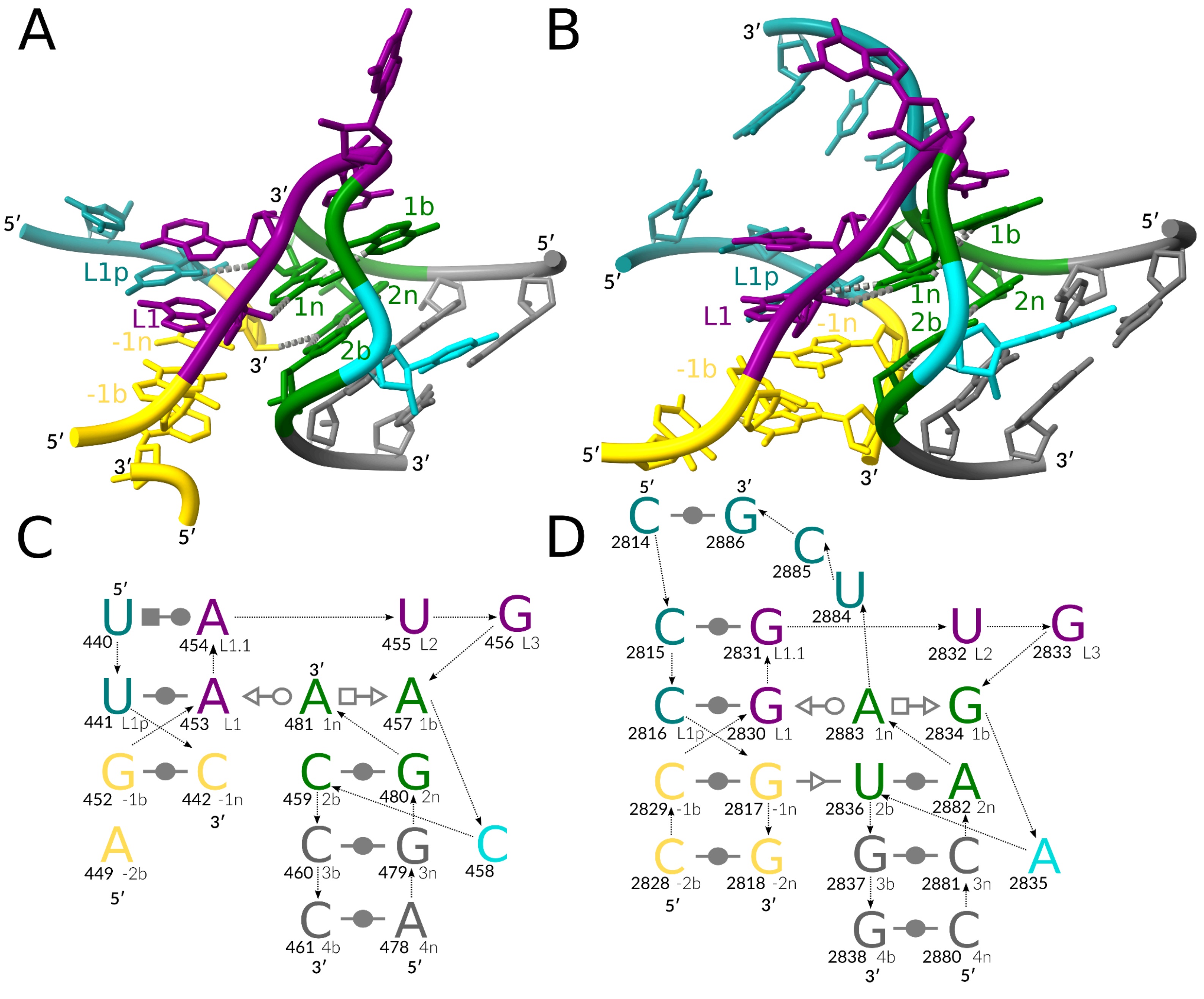

Fig S1. Representative J99/101 k-junction modules. *(A) A 3D structure of the J99/101 k-junction with an external loop architecture, L8 rRNA fragment, PDB entry 7PKT, chain 8. (B) A 3D structure of the J99/101 k-junction with a three-way junction architecture, LSU rRNA, PDB entry 4V8O, chain BA. The interaction schemes of (C) the external loop and (D) the three-way junction: the canonical stem (C-stem) in gold, the*

non-canonical stem (NC-stem) in green and gray, the residues of the kink in purple, the looped-out residues in dark turquoise, and the other residues in teal. The base pair representation follows the Leontis-Westhof (LW) classification [15]. The backbone connectivity is depicted with arrows. 5'- and 3'-ends are shown accordingly. Each base is marked with its number in the chain (in bold, left) and its named position in the kink-turn (right). The 3D representations were prepared with ChimeraX [34], with key residues labeled and prominent key hydrogen bonds indicated with gray dashed lines. The 2D representations were prepared with Inkscape [35].

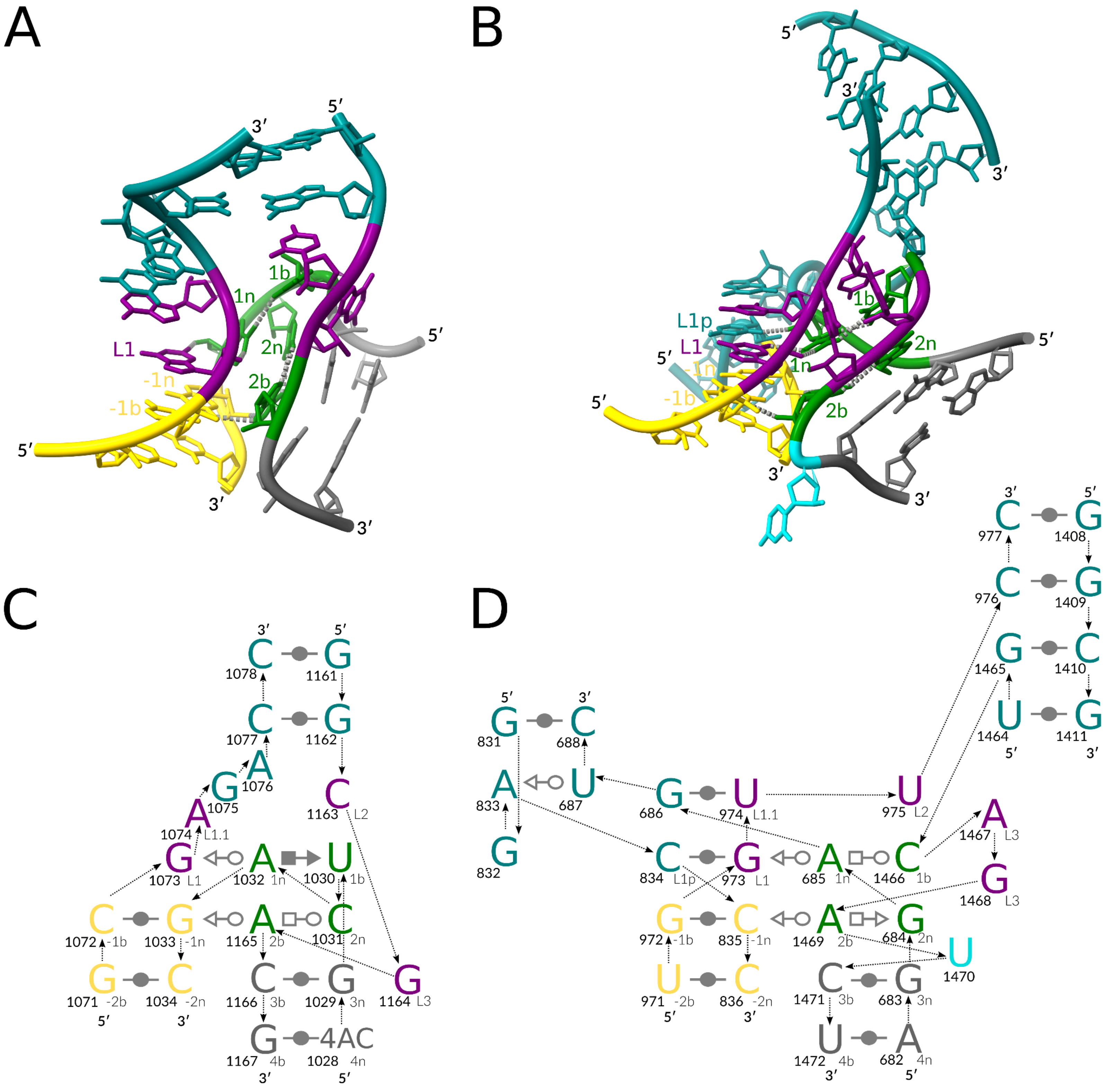

Fig S2. Representative no-kink variants #5 and #6. *(A) A 3D structure of the three-way A-minor junction, 16S rRNA, PDB entry 7ZHG, chain 2. (B) A 3D structure of*

the five-way A-minor junction, 28S rRNA, PDB entry 8EUY, chain 1. The interaction schemes of (C) the three-way junction and (D) the five-way junction: the canonical stem (C-stem) in gold, the non-canonical stem (NC-stem) in green and gray, the residues matching the kink in purple, the looped-out residues in dark turquoise, and the other residues in teal. The base pair representation follows the Leontis-Westhof (LW) classification [15]. The backbone connectivity is depicted with arrows. 5'- and 3'-ends are shown accordingly. Each base is marked with its number in the chain (in bold, left) and its named position in the kink-turn (right). The 3D representations were prepared with ChimeraX [34], with key residues labeled and prominent key hydrogen bonds indicated with gray dashed lines. The 2D representations were prepared with Inkscape [35].

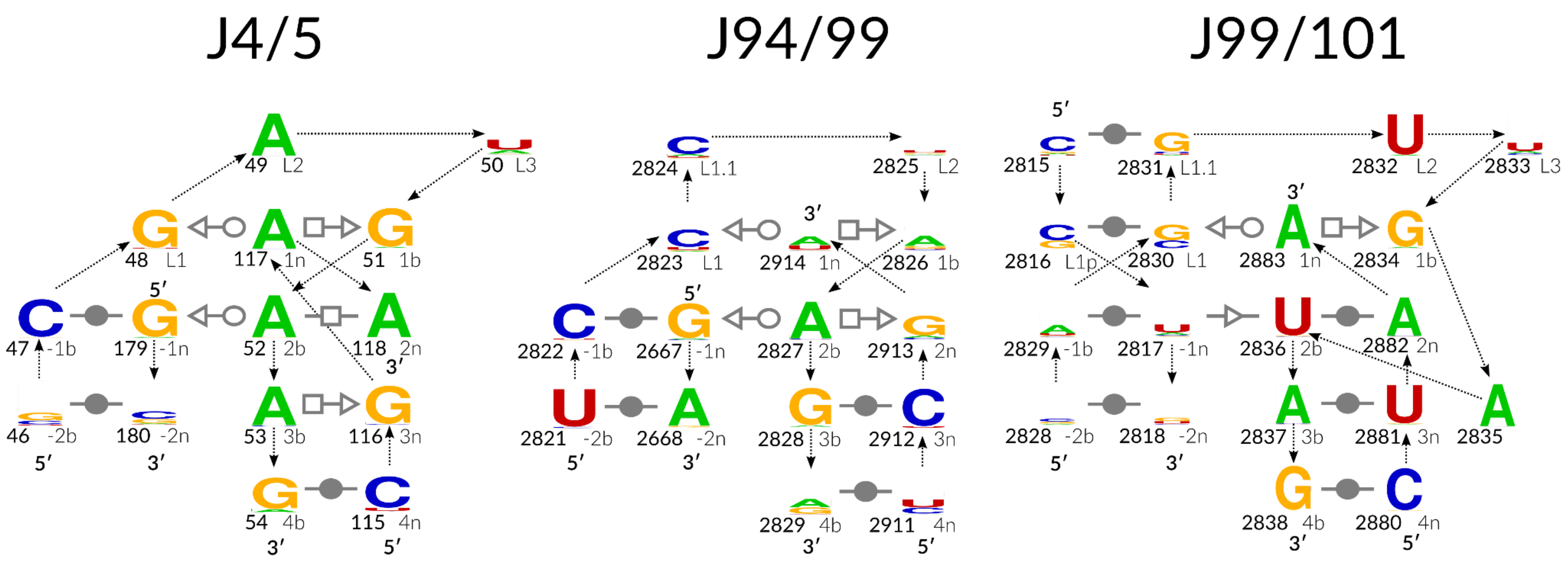

Fig S3. Motif logos of the 23S rRNA k-junctions. *The J4/5 junction logo follows the scheme of module #3, PDB entry 7P7T, chain A. The J94/99 junction logo follows the scheme of module #10, PDB entry 3CC2, chain 0. The J99/101 junction logo follows the scheme of module #9, PDB entry 4V8O, chain BA. The base pair representation follows the Leontis-Westhof (LW) classification [15]. The backbone connectivity is depicted with arrows. 5'- and 3'-ends are shown accordingly. Each base is marked with its number in the chain (in bold, left) and its named position in the kink-turn (right). The 2D representations were prepared with Inkscape [35] and WebLogo [59].*

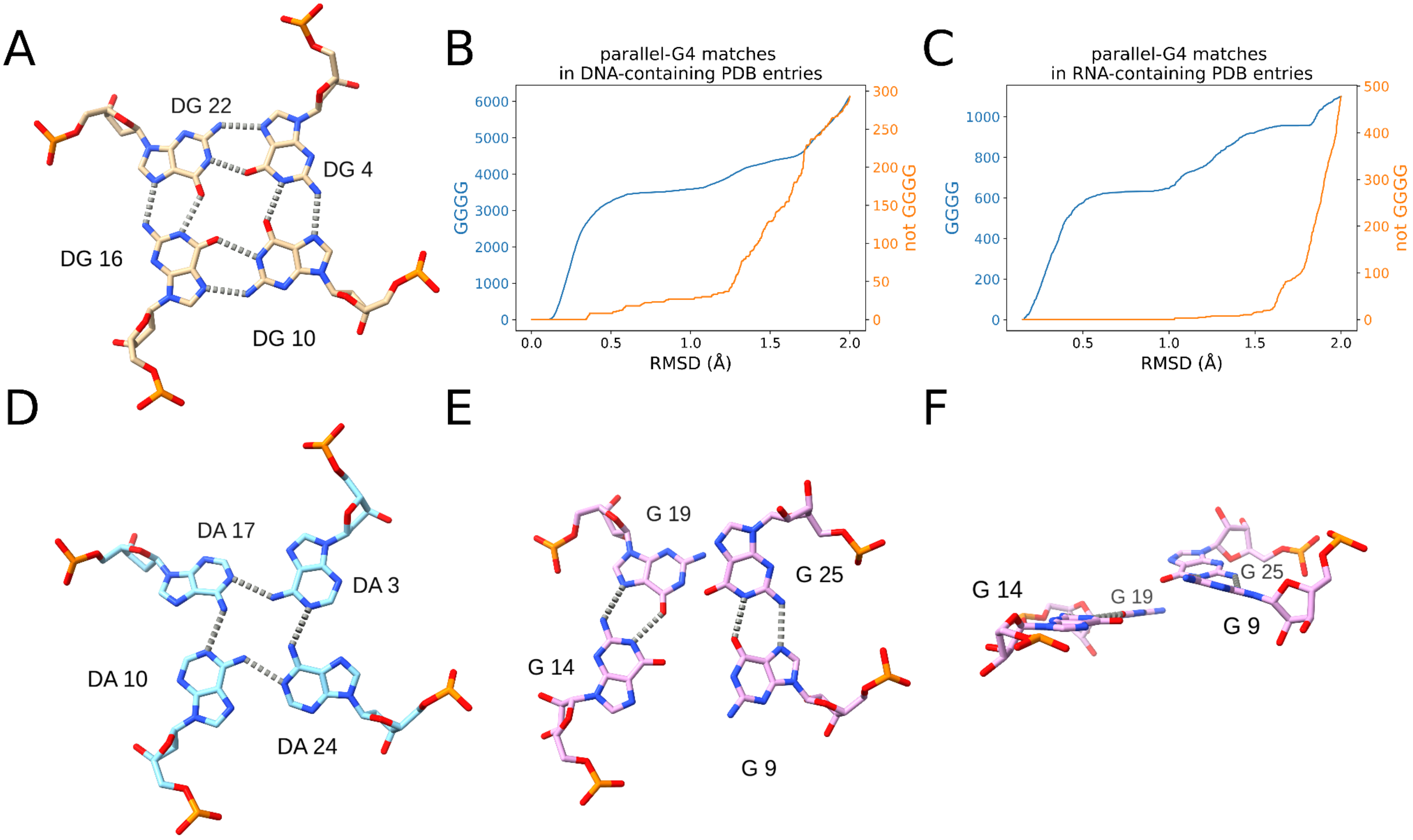

Fig S4. ARTEM search for the parallel G-tetrad motif. *(A) Reference parallel G-tetrad instance from a telomeric G-quadruplex, PDB entry 8D79, chain A. (B) all-G (in blue) and non-all-G (in orange) matches of the motif identified by ARTEM in all DNA-containing and (C) RNA-containing PDB entries. (D) A parallel all-adenine tetrad match identified by ARTEM at RMSD = 0.343 Å in a d[T_2AG_3] DNA repeat tetramer, PDB entry 2JWQ, chains A, B, C, D. (E) Top-view and (F) side-view of a false all-guanine match spanning residues from two adjacent non-parallel tetrads, identified by ARTEM at RMSD = 1.202 Å in an RNA iMango-III aptamer, PDB entry 6E8S, chain B. Prominent hydrogen bonds are indicated with gray dashed lines.*

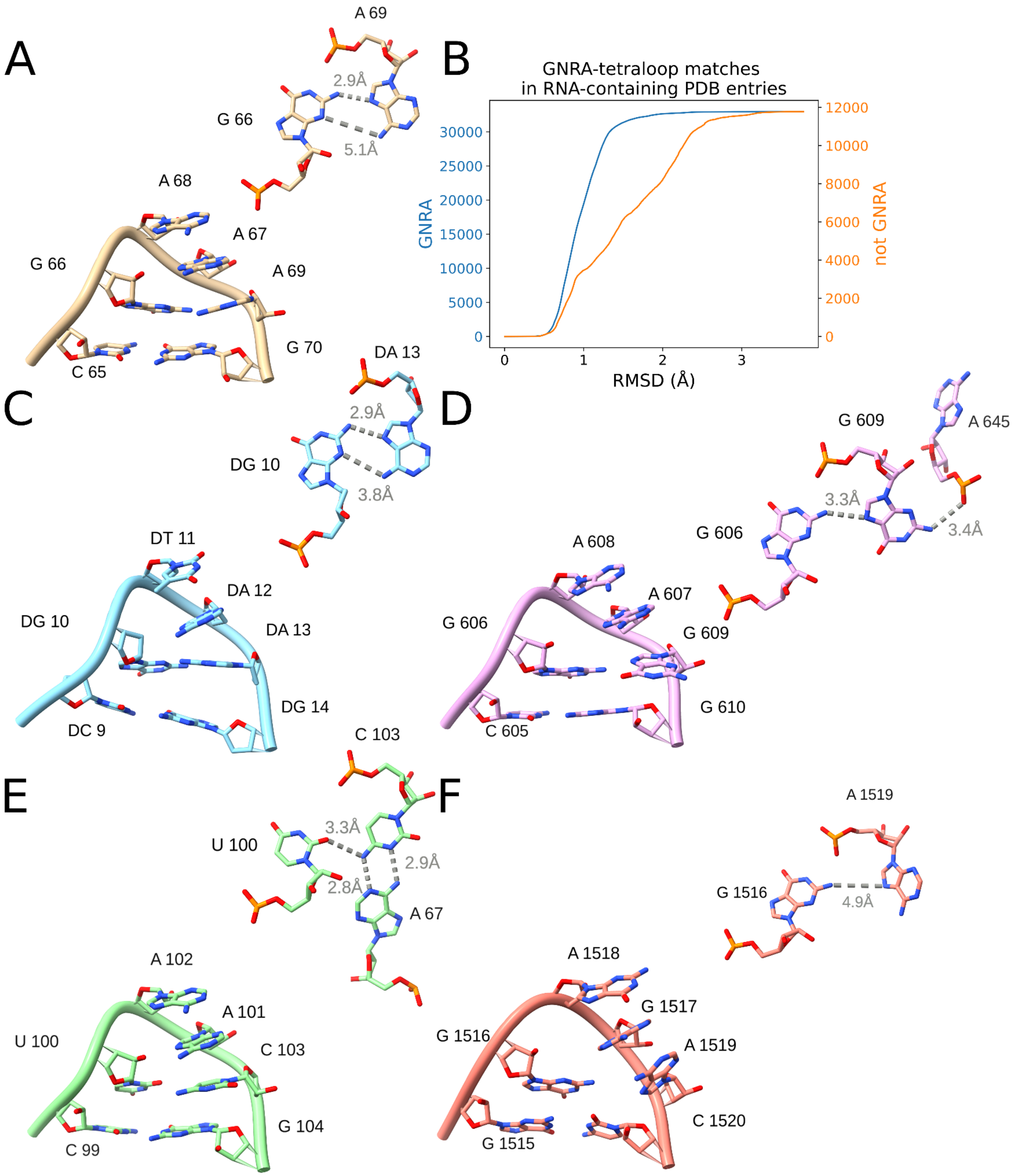
A
A 69
2.9Å
G 66
5.1Å
A 68
A 67
G 66
A 69
G 70
C 65
B
GNRA-tetraloop matches
in RNA-containing PDB entries
GNRA
not GNRA
30000
25000
20000
15000
10000
5000
0
12000
10000
8000
6000
4000
2000
0
0
1
2
3
RMSD (Å)
C
DA 13
2.9Å
DG 10
3.8Å
DT 11
DA 12
DG 10
DA 13
DG 14
DC 9
D
G 609
A 645
3.3Å
3.4Å
G 606
A 608
A 607
G 606
G 609
G 610
C 605
E
C 103
U 100
3.3Å
2.9Å
2.8Å
A 67
A 102
A 101
U 100
C 103
G 104
C 99
F
A 1519
G 1516
4.9Å
A 1518
G 1517
G 1516
A 1519
C 1520
G 1515

Fig S5. ARTEM search for the GNRA-tetraloop motif. *(A) Reference GNRA-tetraloop instance from a THF riboswitch, PDB entry 3SUX, chain X. (B) GNRA (in blue) and non-GNRA (in orange) matches of the motif identified by ARTEM in all RNA-containing PDB entries. (C) GNRA match identified by ARTEM at RMSD = 0.986 Å in a dumbbell DNA, PDB entry 2N8A, chain B. (D) GAAG match identified by ARTEM at RMSD = 0.749 Å in 18S rRNA, PDB entry 7QEP, chain 3. (E) UAAC match identified by ARTEM at RMSD = 0.99 Å in a lariat capping ribozyme, PDB entry 4P8Z, chain A. (F) GNRA match with a distorted tSH G-A base pair identified by ARTEM at RMSD = 1.51 Å in 16S rRNA, PDB entry 6DNC, chain A.*

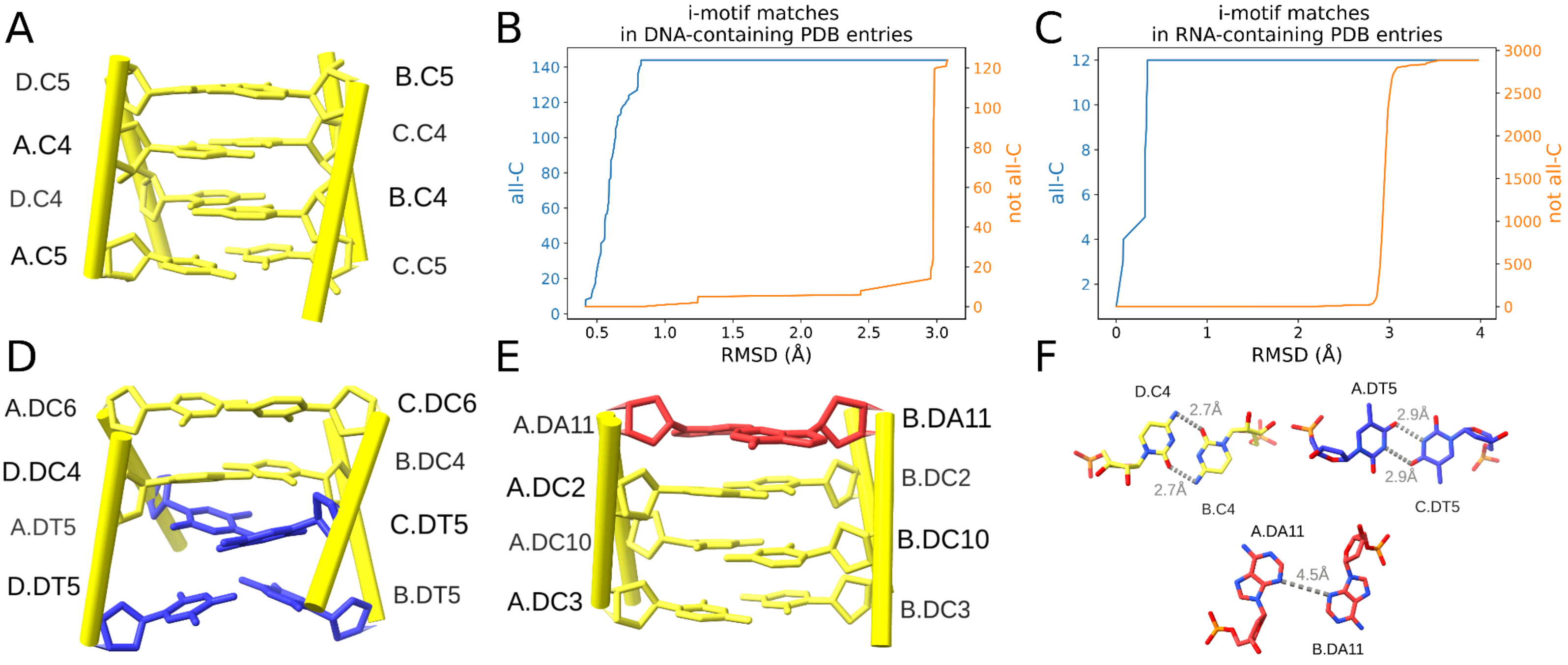

Fig S6. ARTEM search for the i-motif. *(A) Reference RNA i-motif instance, PDB entry 1I9K. (B) all-cytidine (in blue) and not all-cytidine (in orange) matches of the motif identified by ARTEM in all DNA-containing and (C) RNA-containing PDB entries. (D) Thymine-containing match identified by ARTEM at RMSD = 1.004 Å in a DNA i-motif tetramer, PDB entry 2KKK. (E) Adenine-containing match identified by ARTEM at RMSD = 1.24 Å in a double hairpin DNA, PDB entry 1C11. (F) Top view of the tWW C-C, tWW T-T, and tSS A-A base pairs from the identified motifs.*

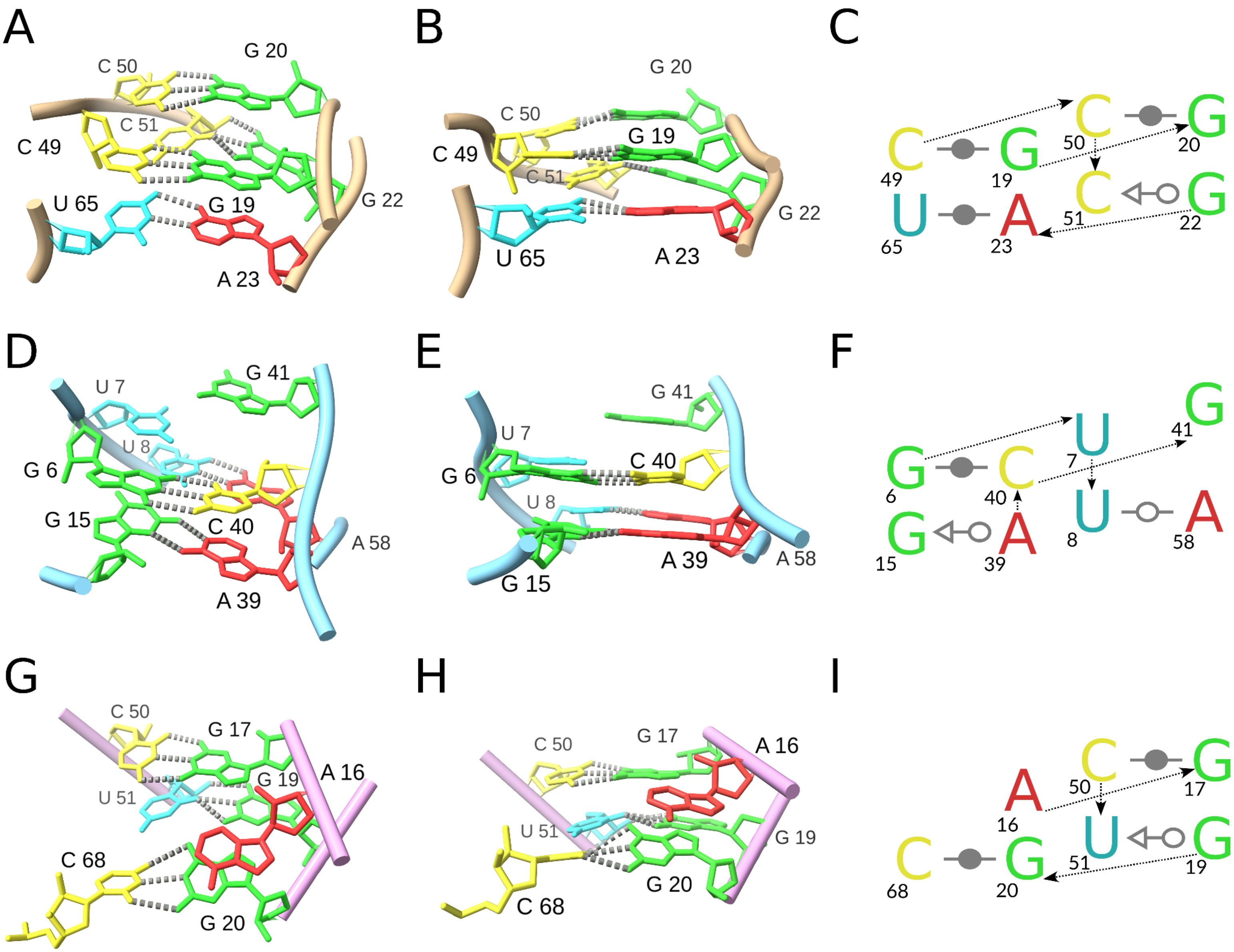

Fig S7. ARTEM search for the parallel-pairing motif. *(A) top view, (B) side view, and (C) interaction scheme of the reference parallel-pairing motif from cap-independent translation enhancers from Pea enation mosaic virus RNA 2, PDB entry 8SH5, chain R. (D) top view, (E) side view, and (F) interaction scheme of the parallel-pairing motif match identified by ARTEM at RMSD = 1.679 Å in a NAD+ -II riboswitch, PDB entry 8GXC, chain A. (G) top view, (H) side view, and (I) interaction scheme of the*

seven-residue parallel-pairing motif match identified by ARTEM at RMSD = 1.19 Å in a SCV CITE RNA, PDB entry 8T29, chain R. Hydrogen bonds are indicated with gray dashed lines.

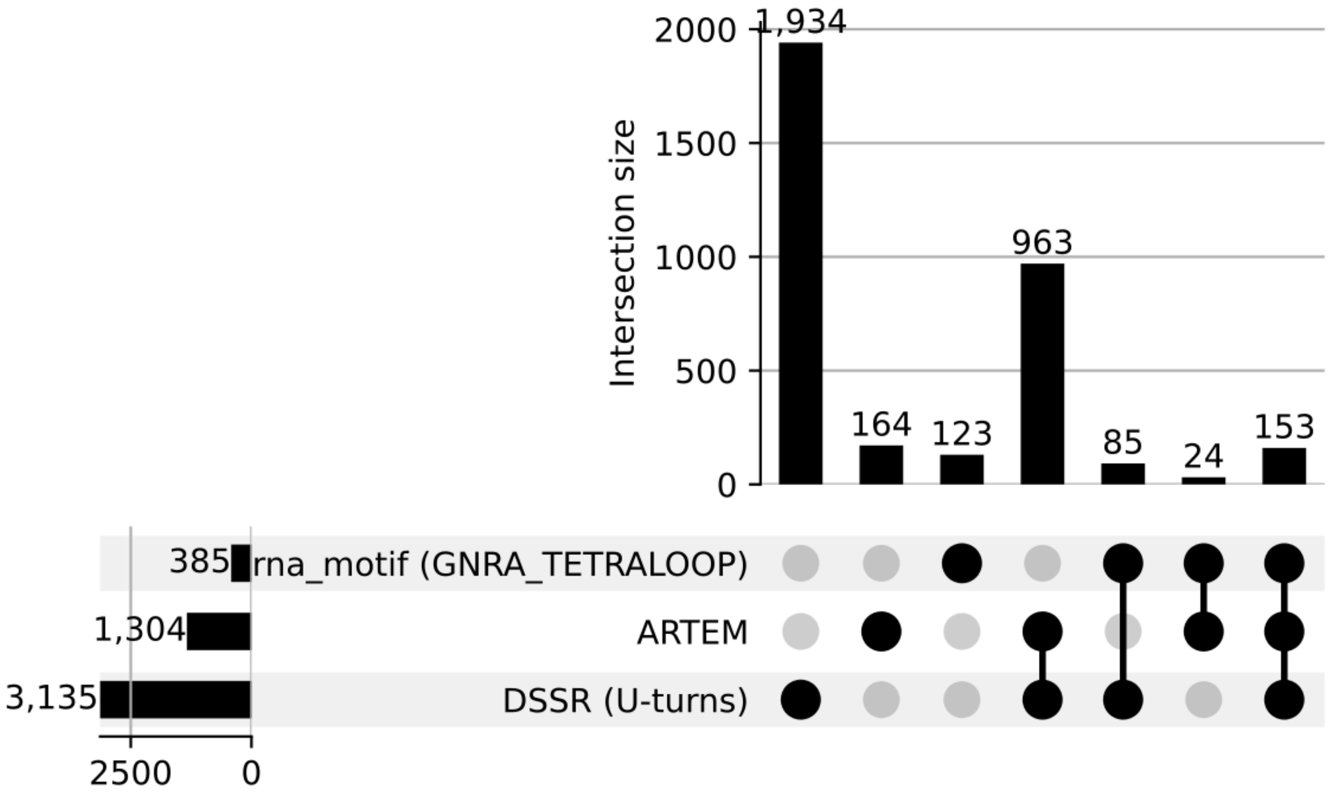

Fig S8. Benchmark of ARTEM, DSSR, and rna_motif Rosetta utility in identifying GNRA tetraloops. *The benchmark was conducted on 2,394 PDB entries from the BGSU representative set of RNA structures. The UpSet plot was generated using the UpSetPlot Python library [37].*

Table S3. Kink-turn characteristics of representative group II intron structures.

PDB entry<br>chain	3IGI<br>chain A	5G2X<br>chain A	6CHR<br>chain A	6ME0<br>chain A	7UIN<br>chain B	8H2H<br>chain A	8T2S<br>chain B
**Intron**	group IIC	group IIA	group IIB	group IIC	group IIC	group IIA	group IIC
**Coordination loop**	C148-C157<br>G220-G226	G203-U210<br>A339-C348	A186-C197<br>G293-U299	A243-C253<br>G362-U368	C151-C160<br>G228-G234	G203-U210<br>A339-C348	C151-C160<br>G228-G234
**L1-1n base pair**	U150-A224(syn)		G188-A297	G245-A366	U153-A232		U153-A232
**Ⲕ extension bulge**	A134-A144	A185-A201	A171-A182	A228-A239	A137-A147	A185-A201	A137-A147
**Ⲕ extension - coordination loop interactions**	tHW<br>A138-U150(L1)<br>stack<br>A137-A151(L2)	stack<br>U195-A344<br>stack<br>U193-G207	ribose-base<br>A175-U189<br>(L1.1)	N4-O6 H-bond<br>C232-G245	tHW<br>A141-U153(L1)<br>stack<br>G140-A154(L2)	stack<br>U195-A344<br>stack<br>U193-G207	tHW<br>A141-U153(L1)<br>stack<br>G140-A154(L2)
**σ-σ′ BWE**				A771<br>A248(L3)<br>A769			
**δ′-δ base pair**	G155-C180		G195-C255	A251-U326	G158-C183		G158-C183
**EBS3**	A223		G296	C365	A231		A231
**IBS3**	A7, chain B		C623	DG15, chain B	DT36, chain A		G1
**Coordination loop residues exposed to reverse transcriptase**				C247(L2)<br>G249(1b)<br>C365(EBS3)	U155(L3)<br>A231(EBS3)		U155(L3)<br>A231(EBS3)
**DIII kink-turn**					G343-U352<br>A366-C372		G343-U352<br>A366-C372

**Coordination loop kink-turn was identified by**	ARTEM ≥12nt<br>ARTEM ≥11nt		ARTEM ≥11nt	ARTEM ≥12nt<br>ARTEM ≥11nt<br>RNAMotifScanX	ARTEM ≥12nt<br>ARTEM ≥11nt		ARTEM ≥12nt<br>ARTEM ≥11nt
**DIII kink-turn was identified by**					ARTEM ≥11nt<br>DSSR<br>RNAMotifScanX		DSSR<br>RNAMotifScanX
